# Bringing the heavy: carbon ion therapy in the radiobiological and clinical context

**DOI:** 10.1186/1748-717X-9-88

**Published:** 2014-03-28

**Authors:** Cody D Schlaff, Andra Krauze, Arnaud Belard, John J O’Connell, Kevin A Camphausen

**Affiliations:** 1 Radiation Oncology Branch, National Cancer Institute, 10 Center Drive Magnuson Clinical Center Room B3B100, Bethesda, MD 20892, USA; 2 Radiation Oncology Service, Walter Reed National Military Medical Center, Proton Beam Program, Building 19- Room B105, 8901 Wisconsin Avenue, Bethesda, MD 20889, USA; 3 The John P Murtha Cancer Center, Walter Reed National Military Medical Center, Bethesda, MD, USA

**Keywords:** Radiotherapy, Radiobiology, Carbon ions, Hypoxia, α/β ratio, Tumor microenvironment, Tumor metabolism, Cancer stem cells

## Abstract

Radiotherapy for the treatment of cancer is undergoing an evolution, shifting to the use of heavier ion species. For a plethora of malignancies, current radiotherapy using photons or protons yields marginal benefits in local control and survival. One hypothesis is that these malignancies have acquired, or are inherently radioresistant to low LET radiation. In the last decade, carbon ion radiotherapy facilities have slowly been constructed in Europe and Asia, demonstrating favorable results for many of the malignancies that do poorly with conventional radiotherapy. However, from a radiobiological perspective, much of how this modality works in overcoming radioresistance, and extending local control and survival are not yet fully understood. In this review, we will explain from a radiobiological perspective how carbon ion radiotherapy can overcome the classical and recently postulated contributors of radioresistance (α/β ratio, hypoxia, cell proliferation, the tumor microenvironment and metabolism, and cancer stem cells). Furthermore, we will make recommendations on the important factors to consider, such as anatomical location, in the future design and implementation of clinical trials. With the existing data available we believe that the expansion of carbon ion facilities into the United States is warranted.

## Towards the establishment of a national ion therapy R&D center

Despite the initial relative success of treatments after the discovery of X-rays in 1895, physicians were left with very few techniques to treat common malignant and benign pathologies that yielded adequate local control (LC) while limiting toxicity and damage to normal tissues and structures [1]. Yet, X-rays were still being used in the clinic without any understanding of their biological characteristics. This lack of understanding unlocked a new and rapidly developing field aimed at comprehending the biological mechanisms of radiation – radiation biology. Clinically, this field focused on the need to achieve better LC, which still remains relevant in modern day radiation therapy (RT) research.

While multiple proton therapy centers are already in operation in the United States, with more under construction, clinical facilities capable of delivering other heavy ions exist notably in Japan and Germany, with more beginning operations or under construction throughout Europe. HIMAC, the Heavy Ion Medical Accelerator in Chiba, Japan, began the first full clinical trials with carbon ion therapy in 1994. HIMAC was joined by two more carbon-beam facilities in 2002 (Hyogo) and 2010 (Gunma). In Germany, the Gesellschaft für Schwerionenforschung (GSI) center has been treating patients with carbon ions since 1997. Preliminary data from those centers suggest carbon ion therapy has the potential to be a superior treatment modality for certain cancer types, but further investigation is necessary. (Discussed below).

In the United States, radiobiology research and clinical treatment using carbon, neon, silicon, and argon ion beams took place from the 1970s to 1993 at the Bevelac, a project at Berkeley’s Heavy Ion Linear Accelerator (HILAC). Worldwide, over 11,000 patients have been treated at heavy-ion facilities [2]. Due to the development and use of heavy ion therapy internationally, and a renewed interest by the Department of Energy to apply accelerator expertise to the medical industry, the ability to conduct carbon ion research may once again become available in the United States [3].

An inter-agency effort to develop and operate a particle beam therapy R&D center at the Walter Reed National Military Medical Center (WRNMMC) in Bethesda, Maryland, was formally launched in August of 2012. This national resource, the only fully operational accelerator-based federal medical research facility in the United States, would be capable of producing ion beams from protons to carbon with the purpose to: 1) serve as a platform for high-quality and high-impact translational, pre-clinical and clinical trials; and 2) operate a fully capable treatment room dedicated solely to radiobiology, medical physics and accelerator physics research and development.

The biggest barriers to clinical research and development of particle beam therapy in the United States for charged particles heavier than protons, are the high capital costs and the high operational costs in the setting of, lack of reimbursements and lack of data demonstrating cost-effectiveness. A zeroth order estimate of the cost of an R&D center in the U.S. is in line with estimates from other groups which have estimated the cost of a center to be on the order of 138.6 million euros [4]. Clearly, securing private investments of this magnitude in order to design, build, and operate a heavy particle R&D center in the United States appears impossible and out of reach especially if investors must wait for effectiveness data to mature [5].

It has been observed in the economic evaluations of proton therapy that in jurisdictions that do not wish to engage in formal reimbursement in the absence of cost-effectiveness data, the introduction of proton therapy may be seriously hampered and will again perpetuate the lack of outcome and cost data [6]. This is even more so in the United States for heavier particle therapy. Pijls-Johannesmaa and colleagues in their assessment of cost effectiveness of particle therapy suggest that other approaches should be considered [5].

An inter-agency collaboration within the U.S. could be one such approach since the agency budgets have virtually no dependence on revenue generated from billing private insurers. Rather cost-avoidance has the potential to provide some savings to federal agencies to offset the costs to their R&D budget, for which many are already spending significantly on cancer research. This together with the well-established national healthcare systems of the various U.S. federal agencies, the large numbers of eligible beneficiaries they care for, the high quality of cancer care that they deliver, and the proven ability of multiple federal agencies (e.g. the National Cancer Institute’s Center for Cancer Research and the Veterans Administration Office of Research and Development) to design and conduct high impact clinical oncology trials on a national scale make a federal inter-agency effort in collaboration with academia and industry an approach. Grutters, *et al*., astutely observe that postponing the decision to adopt a potentially cost-effective treatment induces costs in terms of health benefits forgone [7]. In the case of particle therapy for the treatment of non-small cell lung cancer (NSCLC) they assert that because of the high value of information, it is recommended to acquire more evidence on the effectiveness of particle therapy in NSCLC. However, they point out that collecting clinical evidence requires particle facilities. They therefore conclude that, it might be worthwhile to invest in a particle facility, which should initially be used for clinical research only [7]. We agree and believe that investigators in the U.S. would have much to contribute to this important research.

Following the recommendations of the Summary Report - Workshop on Ion Beam Therapy, the proposed R&D center would exist to advance both research and treatment options for tumors a) exhibiting a high-risk of local failure post photon (or proton) RT, b) radio-unresponsive due to histology, hypoxia, and other factors, c) recurring, d) efficient at repairing cellular damage, or e) adjacent to critical normal structures, especially if resection could lead to a substantial loss of organ function [8].

In this review, our aim is to discuss how radio- and tumor biology, anatomical factors, and other non-classical mediators of photon-therapy resistance should be taken into account when optimizing the use of carbon ion therapy for cancer management. Additionally, we will provide recommendations for the design of future clinical trials, and recommend which malignancies could be initial primary targets for the introduction of carbon ion therapy into mainstream clinical practice. It is worthwhile to mention that the use of other high LET particles have been used for decades (e.g. fast neutron therapy) or are current candidates for therapeutic use (helium and oxygen), in many of the same histologies that are discussed here; however, this comparison is beyond the scope of this review. Many of the same conclusions drawn here may also be valid, to a lesser degree, for other heavy ion particles. Future work should be done in analyzing dose and fractionation schemas implemented with fast neutrons to help determine dose settings in future phase I/II carbon ion clinical trials.

### Radiobiological factors

It is tempting to present carbon ion technology as a valid option for most malignancies based on a variety of radiobiological parameters. Considering the higher relative biological effectiveness (RBE) and increased linear energy transfer (LET) carbon ions possess, they should theoretically produce greater outcomes for any malignancy for which they are being employed. However, multiple reasons exist for the selective use of carbon ions including, the significant cost associated with construction and operation of facilities, and moreover, the lack of long term data regarding toxicity and secondary malignancy.

According to Fokas *et al*., high LET radiation should be selectively used for radiobiological reasons in tissues that are: slowly proliferating, later responding, have a high capacity for sub-lethal damage repair (SLDR), a low α/β ratio, and in those histologies which have been shown to be highly resistant to conventional treatment [9]. This statement challenges the balance of high RBE for carbon ions, against radiobiological and anatomical factors. This dichotomy limits its actual implementation for many malignancies. In this section, we will discuss the classical radiobiological factors that play critical roles in determining which malignancies should theoretically respond well to carbon ion RT.

### The α/β ratio

A hallmark component of classical radiobiology, the α/β ratio, is one of the overriding parameters used to model cell killing by radiation. It is the byproduct of the linear quadratic (LQ) model, which describes cell killing as a single hit versus double hit hypothesis, where linear cell kill is expressed by the α component, while quadratic cell kill is expressed by the β–component [10]. The ratio is obtained from isoeffect curves plotted using the survival fractions (SFs) of a single cell line at different doses per fraction [11,12]. Presently, this ratio is used as a staple for predicting the clinical effects in response to RT despite various limitations.

A high α/β ratio (6–14 Gy), seen in most human tumors, suggests a predominance of the α-component, implying a decreased response to fractionation and therefore, clinical benefit from hyperfractionation. (Hyperfractionation is implemented in order to spare normal tissues, prevent accelerated repopulation, and maximize therapeutic gain). A lower α/β ratio (1.5–5 Gy) is usually associated with late responding normal tissue, and is the basis for the therapeutic gain achieved using hypofractionation. However, some tumors have been postulated to have a low α/β ratio, including prostate cancer, rhabdomyosarcoma, and melanoma [13,14].

**Figure 1:**
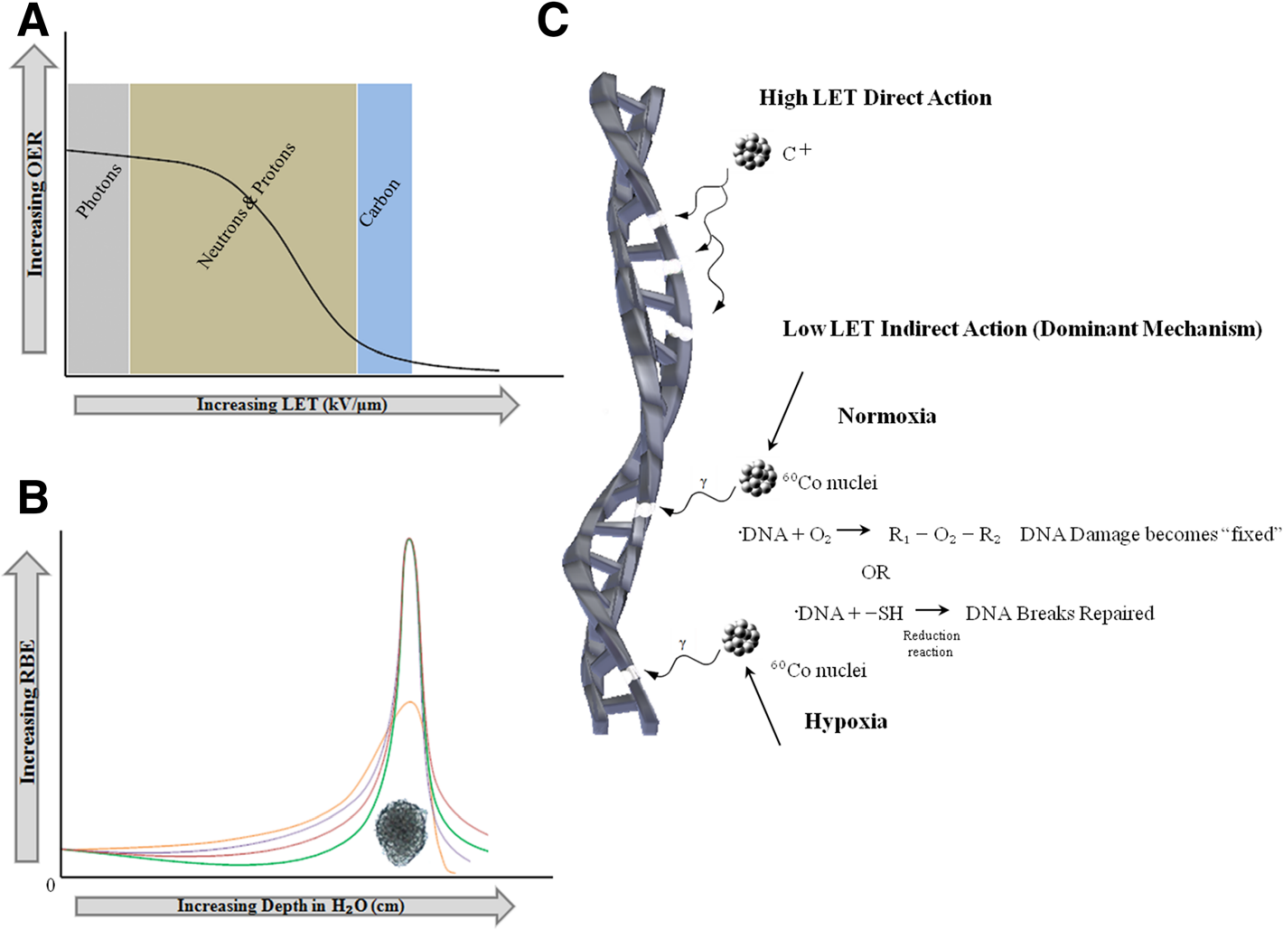
**Radiation Species determine the importance of the classical radiobiological factors. (A)** The oxygen enhancement ratio (OER) has an inverse relationship with the linear energy transfer (LET). While the cell killing effect of photons (grey) and protons (brown) are dependent on the oxygen tension, carbon ions (blue) are able to induce the same cell kill effect with a significantly lower degree of dependence on oxygen tension. **(B)** The attractiveness of protons (orange) and other heavier ion species, such as, neon (red), helium (purple) and carbon (green) is the existence of the Bragg Peak, which allows for minimal damage to the surrounding tissue, while low LET radiation, which does not exhibit this peak can induce greater damage to the surrounding tissue. Carbon ions have become a more popular option as it has the lowest entry RBE of other heavy ion species, and unlike protons, does exhibit fragmentation tails intermediary of the other heavy ion species, however more importantly, at the Bragg Peak has a significantly higher RBE compared to protons. **(C)** Under normoxic conditions low LET photons hydrolyze water and induce breaks in the phosphodiester bonds of DNA. Subsequently the DNA radicals in the presence of molecular oxygen will be fixed or become permanent. Under hypoxic conditions, however, the DNA radical becomes reduced by sulfahydrl groups and the DNA breaks become repaired. With high LET radiation (carbon) the particle directly acts on the phosphodiester bond of DNA inducing clustered damage which is less amenable to repair.

In theory, a possible rationale for the administration of carbon ion therapy can be successfully argued for both high and low α/β tumors. For low α/β tumors, carbon ions could eliminate the relative radioresistance to photon treatment, by decreasing the predominance of the β-component, and subsequently decrease the capability for SLDR. Sublethal damage is typically associated with photon irradiation. By contrast, carbon ions tend to cause “clustered” damage, which is less prone to SLDR, and accordingly, may potentially increase the LC of low α/β ratio tumors [15,16]. High α/β tumors on the other hand, already tends to show a more robust response to photon irradiation by virtue of their high α component. Yet, like low α/β tumors, they too can derive theoretical benefit from carbon ion treatment twofold: (1) increased cell killing beyond what is achieved by photon RT, as a result of superior RBE, and (2) a decrease in toxicity to normal tissue due to the superior depth dose distribution of carbon ions (Figure 1).

Examination of the α/β ratio of various tumors is necessary to utilize this ratio in guiding the selection of tumors. Yet, these data are not widely available for a variety of common human tumors due to, significant concern over its determination in cell lines (some of which may be inadequate), the influence of the tumor microenvironment *in vivo*, and the difficulty accounting for hypoxia. Where available, it has been obtained from experimentally derived tumors irradiated and assayed *in situ* by growth delay [14]. An alternative to the α/β ratio is to look at the surviving fraction at 2 Gy (SF2) of various tumors, as a surrogate for the radiosensitivity of photon irradiated tumors.

Deacon *et al*. classified tumors into 5 categories A to E according to radioresponsiveness based on the SF2, with A being the most radioresponsive and E the most radioresistant [17]. Fitting the LQ equation to the mean SF2’s correlated with a α/β ratio of 60.4 for group A and 5.77 for group E. Not surprisingly, tumors identified by an elevated SF2, such as category E tumors (glioblastoma, melanoma, osteosarcoma, and renal cell carcinoma) remain difficult to control using photon RT. Based on multiple experimental findings, this particular set of tumors maintain an increased ability for SLDR, and have a wide α/β ratio range, thereby exhibiting relative radioresistance to photon irradiation.

Expectedly, the category E tumors that have been treated with carbon ions have responded with promising results. Mucosal malignant melanoma treated with carbon ion RT in conjunction with DAV chemotherapy gave a survival rate of 58%, similar to the survival rate with post operative photon RT or carbon ion therapy alone, 51.5% and 35% respectively [18,19]. Bone and soft tissue sarcomas of the head and neck, another category E tumor, specifically when unresectable, were shown to have a 5-year LC rate of 73% and a 5-year overall survival (OS) rate of 48% [20]. When using photon RT alone, these tumors have a LC rate of only 43-50%.

Prostate cancer, another category E histology that to date, has been increasingly treated with carbon ions has shown success, although its treatment with carbon RT may not be necessary [21]. Photon and proton therapy have been the standard of care and have shown success [22-24]. The argument to use carbon ions for this malignancy is to decrease the risk of potential side effects that can be encountered with photon RT due to the superior depth dose profile of carbon ion treatment. In addition to this, hypofractination, which is often employed in carbon ion treatment, could improve patient convenience.

Previous trials suggest toxicity similar to or better than proton therapy, with slightly improved OS in higher risk groups [21]. These findings are not surprising as they adhere to current radiobiological thought. It is likely that the higher risk prostate cancers are more radioresistant (low α/β and SF2), and therefore, more likely to benefit from carbon ion treatment. Considering the SF2 and α/β ratio, low and intermediate risk prostate cancers may derive equal benefit from both proton and carbon RT.

This observation fuels the argument that the use of carbon ions may improve LC in other category E tumors. Interestingly, little literature is available on the α/β ratio or SF2 on chordomas (α/β ratio: ~2.45), chondrosarcomas or adenoid cystic carcinomas, however, these malignancies are some of the primary malignancies treated with carbon ions in a study that was terminated at GSI [25,26]. Presumably, these tumors were chosen because of a high local failure rate when given photon irradiation. Additionally, anatomical considerations (discussed below), rather than the availability of these data, may provide support for using carbon ions.

Exploiting the α/β ratio, however, requires further study, and in the absence of long term data, carbon ions should not preferentially be employed in high α/β tumors outside of a clinical trial, unless significant retrospective evidence exists producing a superior outcome compared to photon RT.

### Carbon ion RBE

RBE is not only cell type dependent, but also varies with particle energy (Figure 1). The carbon ion energy distribution over the treatment field is inhomogeneous, and therefore, the ability to accurately predict RBE at various dose depths and tissues will be crucial in eliciting a therapeutic advantage over photon treatment [27]. The RBE of carbon ions is optimal at the Bragg Peak, but the Bragg Peak is also tissue dependent. Data on the RBE with respect to different tissue types is emerging; however, it is not currently being employed directly in treatment planning. By not fully using available experimental RBE data in conjunction with different tissue types, the risk of neglecting to identify the presence of a clinically significant effect when carbon ions are incorporated in clinical trials exists.

### Hypoxia

The concentration of oxygen and its effects on radiosensitivity have been meticulously investigated since the early twentieth century, beginning with Petry in 1923, where the observation was made that radiation inhibited the germination of vegetable seeds [10]. This oxygen effect was further confirmed through the quantitative measurement of oxygen on growth inhibition of the *Vicia faba* primary root [28]. Exhaustive research has since gone into understanding this oxygen effect, as the absence of oxygen is postulated to play a role in conferring radioresistance in tumors. Investigators have now unequivocally demonstrated the effect that the absence of oxygen has on radioresistance, and the negative effects it has on tumor control, yet this observation cannot be fully explained by radiobiology or physics [29-31].

Experiments have shown that at both low and high doses of radiation, an enhancement of cell killing in aerated conditions is observed when compared to hypoxic conditions thus giving rise to the concept of the oxygen enhancement ratio (OER). Fascinatingly, minimal oxygen effect has been observed for densely ionizing radiation [10]. This may be explained by the complexity of DNA damage that intermediate and densely ionizing radiation (e.g. α-particles, carbon ions) are capable of inducing on DNA [32] (Figure 1).

Following exposure to ionizing radiation, if molecular oxygen is present, organic peroxide is produced, thus “fixing” or making permanent the damage incurred by DNA. Under hypoxic conditions however, DNA damage induced by low LET radiation can be more readily repaired. The DNA radical can be reduced by sulfahydryl groups (SH groups) making DNA damage, both single and double strand breaks, less severe under hypoxic conditions. A related explanation may be that the fixation of DNA damage by oxygen could be relevant for indirect radiation effects, the dominant form in low LET radiation, while direct action caused by high LET radiation (e.g. carbon) is less affected by the presence of oxygen [33] (Figure 1). Numerous alternative hypotheses try to explain this phenomenon, however, as of yet, there is no uniform theory that is capable of explaining the inverse relationship between OER and LET [34-37].

Increased exploration showed that there is a very complex correlation between the tumor microenvironment and the significant heterogeneity in the pathways that govern the response to hypoxia in different tumors. Oxygen tension is known to be quite heterogeneous in tumors with many regions having very low levels, much lower than in surrounding normal tissues (in some tumors less than 5 mmHg pO_2_) [38]. Studies have shown an inverse relationship between dependence on oxygen inducing cellular damage and the mass of the ion species. Consequentially, one would expect tumors with larger hypoxic fractions to benefit from carbon ion radiation.

A theoretical analysis by Wenzl and colleagues determined that dose dependence existed between OER and the dose per fraction given [39]. They determined that the behavior of the OER depended primarily on the α and β parameters which in turn depend on LET, pO_2_, and cell or tissue type. In comparing multiple studies of various cell lines they observed that the α, α_*aeorbic*_/α_*hypoxic*_, and β, (β_aerobic_/β_hypoxic_)^1/2^ components were in general, substantially lower when exposed to high LET carbon radiation, than those cell lines that were exposed to low LET radiation.

The identification of tumors that are radioresistant by virtue of hypoxia may offer a rationale for these tumors to be targeted with carbon ion RT. Since it is difficult to measure the α/β ratio in tumors, the measurement of a hypoxia biomarker could lend additional information in determining the therapeutic gain when deciding to pursue carbon ion irradiation.

**Figure 2:**
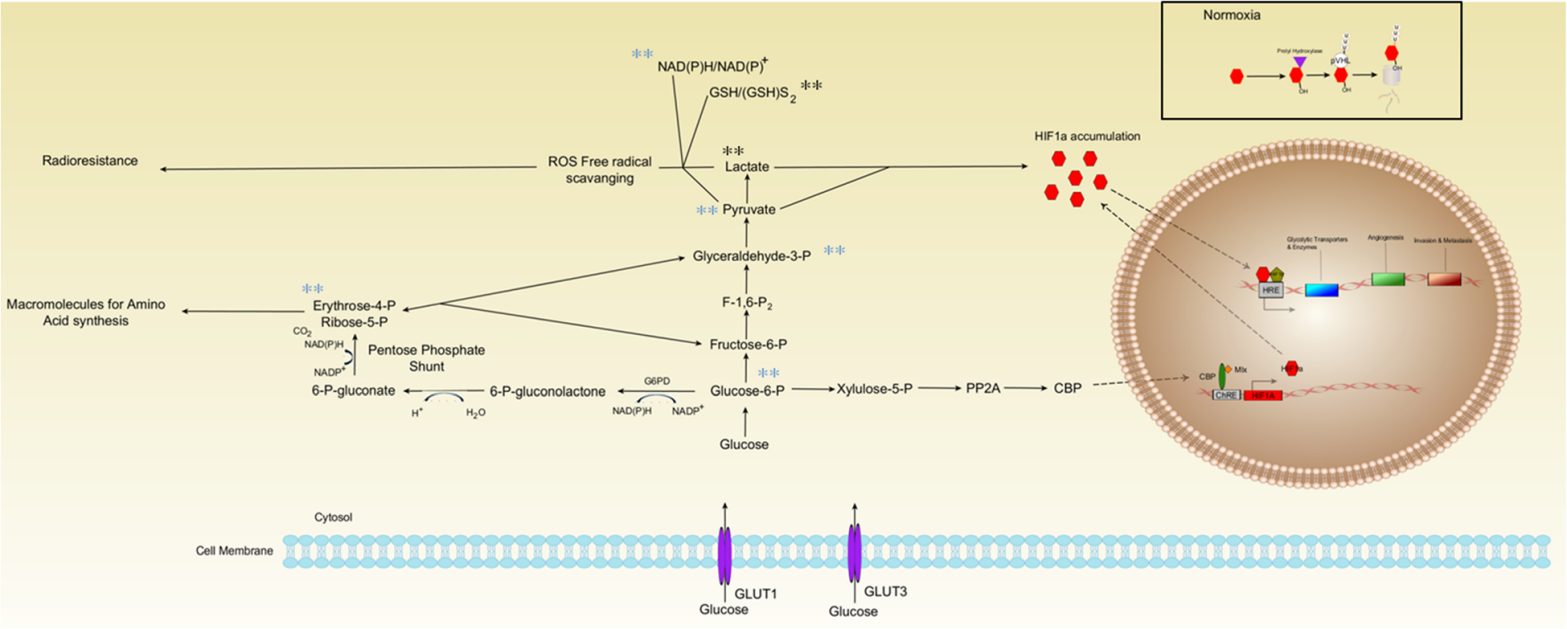
**The interplay of the tumor microenvironment on radioresistance and glucose metabolism.** Under normoxic conditions (insert) HIF1α is targeted for degradation. The ODD domain is hydroxylated and ubiquinated by prolyl hydroxylases (purple triangle) and pVHL-containing E3 ubiquitin ligases (circle). However, under hypoxic conditions HIF1α is stabilized and activated by binding to HIF1β and the resulting dimer binds to the HRE inducing the expression of genes leading to angiogenesis, such as VEGF and SDF1, invasion, metastasis, and glycolytic transporters and enzymes (GLUT1). Furthermore, the cells use of the inefficient ATP producing glycolytic pathway may affect hypoxic and normoxic radioresistance. Key intermediaries of the glycolytic pathway, glucose-6-phosphate, pyruvate, lactate, and the reducing couples NAD(P)H/NAD(P)^+^ and GSH/GSH-disulfide, have been observed to play roles in continuing the cycle of maintaining HIF1α. Glucose-6-phosphate can either enter into the pentose phosphate pathway leading to the synthesis of erythrose-4-phosphate and ribose-5-phosphate which are necessary for amino acid synthesis or can feed back into glycolysis and create lactate and pyruvate, which lead to HIF1α accumulation continuing the cycle of transcription. Alternatively, glucose-6-phosphate can also lead to the transcription of HIF1α by entering into the pathway which ultimately leads to the nuclear translocation of CREB-binding protein (CBP) which binds with Mlx leading to HIF1α transcription. Lactate and pyruvate along with the reducing couples scavenge reactive oxygen species (ROS) free radicals which can also lead to radioresistance. It is still unclear whether radiosensitizing drugs are necessary for carbon ions; however some experiments have shown that targeting specific crucial players of the glycolytic pathway in combination with carbon ions leaded to enhanced cell kill. It may warrant targeting other important intermediaries (i.e. glucose-6-phosphate) with carbon ions to possibly enhance treatments. Black asterisks represent experimentally determined radiosensitization; blue asterisks represent hypothesized radiosensitization targets.

Identifying hypoxia induced genes and downstream signaling molecules associated with radioresistance may help in determining which types of tumors would benefit from carbon ion RT. A plethora of evidence has shown the increasing importance of the heterodimeric transcription factor, hypoxia-inducible factor 1 (HIF-1) [40]. Expression of the α-subunit has been observed to correlate with a poor prognosis, local recurrence and distant metastases subsequently following irradiation. (reviewed in [41]). In short however, the most characterized mechanism is that under normoxic conditions, the oxygen-dependent degradation (ODD) domain is hydroxylated and subsequently ubiquinated by prolyl hydroxylases and pVHL-containing E3 ubiquitin ligase respectively, leading to the degradation of HIF-1α. While, under hypoxic conditions, HIF-1α is stabilized and activated, binding with HIF-1β, and the resulting HIF-1 protein binds to the hypoxia-responsive element (HRE) inducing the expression of genes leading to angiogenesis, invasion and metastasis [41] (Figure 2).

Therefore, one possible model to explain hypoxic cellular radioresistance is that vascular endothelial growth factor (VEGF) expression is induced by the activation of HIF-1. VEGF has been shown to protect endothelial cells from the cytotoxic effects of radiation allowing these vessels to supply oxygen and nutrients to the tumor cells, promoting growth [42]. Alternatively, hypoxia induces the upregulation of survivin in tumor cells, a protein belonging to a family known to inhibit apoptosis, and play crucial roles in this pathway, as well as, cellular division. Reports have shown that the expression of survivin correlates with the radioresistance of pancreatic, colorectal and lung cancer cells, and siRNA knockdown of this target enhanced radiosensitivity [43]. These two models may work in conjunction with each other since a copy of the HRE element is present in the core promoter of survivin and its expression has been shown to correlate with HIF-1α expression [44-46].

The actual measurement of hypoxia in tumors has proven to be difficult, and to date there is no single standard method for its measurement. Popular methods include pO_2_ electrodes, immunohistochemical (IHC) detection of injected drugs, the IHC detection of proteins that are overexpressed in hypoxia, such as HIF1α, and imaging techniques involving hypoxic cell radiosensitizer molecules via positron emission tomography (PET), and magnetic resonance imaging (MRI) [47]. Certain measurement modalities, like pO_2_ electrodes, can only be applied to superficial tumors such as melanoma or cervix cancers due to the invasiveness of the procedure. However, these electrodes have been used invasively in non-superficial tumors such as GBM [48-50].

Nordsmark *et al*. 2005 has shown that a high degree of hypoxia (defined by the 2.5 mmHg pO_2_ level) was significantly linked to treatment failure in an overview of 397 head-and-neck cancer patients from seven centers [51]. Cervix cancer has also been associated with increased prognostic relevance of pO_2_ and a pO_2_ of 2.5-10 mmHg has been associated with decreased LC [47,52]. In the case of glial brain tumors, hypoxia imaging has been wrought with difficulties, with an inability to distinguish whether nitroimidazole staining is prognostic or merely indicative of tumor grade [53-55].

Thus, if we were able to identify the sets of patients in whom hypoxia is responsible for radioresistance, this could be exploited with carbon ion treatment, and the possible addition of radiosensitizing agents. Radiosensitivity and resistance are multi-factorial, and a simple relationship between tumor hypoxia and radioresistance is unlikely. Solely measuring the oxygen concentration in the tumor is unlikely to help select those tumors best suited for carbon ions; as the relationship between radioresistance and hypoxia is far more likely to be a complex one not defined by this one factor alone.

### Cell cycle dependency and accelerated repopulation

The position of cells in the cell cycle has been shown to be seminal in determining radiosensitivity [56]. Copious literature exists illustrating that cells are most sensitive to photon irradiation in the G2/M phases of the cell cycle, and most resistant in late S phase [10]. This increased radiosensitivity in G2/M appears to be related to chromatin condensation and thus the effective repair of DNA damage is less likely, due to the inability to perform homologous recombination in the absence of a complementary DNA strand [57]. Unlike low LET radiation, the distribution of cells in the cell cycle has no significant effects on radiosensitivity when employing high LET radiation. However, preclinical studies have suggested that S-phase specific radiosensitivity may exist with high LET radiation [58]. If this observation holds true, it would further bolster the argument that carbon ion treatment can find a niche in low LET resistant tumors.

But, since carbon ions exhibit less cell cycle dependency, this could potentially result in increased cell kill of both slowly proliferating tumors and normal tissues, which could decrease therapeutic gain. Conversely, the ability to exploit molecular triggers for apoptosis in some cell types (e.g. the ability to induce p53-independent apoptosis) could create a larger therapeutic window by taking advantage of the superior dose depth distribution of carbon ions, and decrease the impact of accelerated repopulation in rapidly cycling tumors in the absence of hyperfractionation [59].

At the beginning stages of treatment a majority of tumor cells may lie quiescent, thereby being more radioresistant. As the tumor begins to shrink, the surviving clonogens undergo accelerated repopulation, rapid division, ultimately leading to local failure [60]. This observation has prompted accelerated dose delivery, using fractionation schemes that reduce the overall treatment time to minimize the impact of repopulation. Unlike low LET radiation, carbon ions, due to cell cycle distribution independence, could overcome accelerated repopulation without the need for accelerated treatment regimens.

### Tumor cell proliferation

The ability for tumors to proliferate can be expressed in their potential doubling time (T_pot_), volume doubling time, Ki-67 index, or presence of mitotic figures [10,61-63]. While rapidly cycling cells may be initially more responsive to photon irradiation, long term, they are more likely to recur locally. The relationship between proliferation rate and resistance to photon irradiation is particularly strong in head and neck, and lung tumors [64].

Similarly, squamous cell carcinoma (SCC) of the head and neck tends to exhibit rapid proliferation rates and radioresistance with an elevated local recurrence rate with photon irradiation. The possibility of accelerated repopulation in between photon fractions due to their new found access to oxygen, and their rapid proliferation rate, has prompted manipulation of the fractionation schedule for photon treatment with hyperfractionation (i.e. accelerated hyperfraction and CHART). A study by Fowler and Lindstrom found that with prolonged RT there was a 12% average loss of LC per week [65]. The disadvantages have been the organizational difficulties in carrying out such schedules, as well as, the acute reactions experienced by the patient. If a LC benefit becomes realized with carbon ions, a carbon ion treatment schedule would both offer improved outcomes and patient convenience, and alleviate the need for hyperfractionation to counteract accelerated repopulation.

We have provided indirect evidence that carbon ion RT may overcome low LET radioresistance, however it is valuable to emphasize that the radiobiological factors at work may differ from tumor to tumor, and even among patients. Furthermore, in some tumors, resistance to low LET irradiation could be secondary to complex molecular switches some of which have yet to be identified. Despite the radiobiological benefits of carbon ions, the potential exists that their use may eliminate the therapeutic gain between tumor and normal tissue that may have historically been exploited using low LET RT regimens. As a result, carbon ions should initially be used in malignancies where using conventional photon irradiation proves ineffective. The loss of therapeutic gain can be offset by the decreasing amount of normal tissue irradiated due to the superior depth dose distribution of carbon ions.

### Neo-radiobiologic factors: lack of response to photons - beyond classical radiobiology

Having discussed the classical aspects of intrinsic radioresistance to low LET radiation, and how carbon ions can be employed to exploit them, other factors such as, the molecular aspects of tumor biology and its microenvironment have recently become of interest. In fact, these factors may even render some tumors radioresistant above and beyond the classical contributors to radioresistance. Some of the current interests include: the presence of cancer stem cells (CSCs), the tumor microenvironment, and metabolism. The presence of adaptive radioresistance will also be discussed in this context. It is our intention to show that these other factors may no longer confer radioresistance when treating with carbon ions.

### Presence of stem cells

The discovery of CSCs and the discussion of a potential hierarchical model, wherein only a subset of tumor cells within the tumor bulk may possess the capacity to regenerate have given rise to multiple avenues of research aimed at the eradication of this cell population to improve clinical outcome [66,67]. Incidentally, they have been shown to be chemo- and radioresistant, as compared to their well differentiated counterparts [10]. Additionally, CSCs may be aiding in the maintenance of a tumor microenvironment (a low pH, hypoxic and nutrient deprived environment) increasing the likelihood of radioresistance to photons [10].

Solid tumors that have been shown to possess a CSC subset consequentially include a significant proportion of tumors previously identified as being radioresistant to photon RT [68-73]. Interestingly, the very factors that confer radioresistance in cancers cells (i.e. hypoxia and nutrient deprivation) are the very same that promote the growth of CSCs [74-76]. Moreover, it has also been shown that treatment with low dose photons increases the proportion of radioresistant stem cells (radioadaptive resistance) [77,78]. Repopulation, as one would potentially expect to occur with photon irradiation, has also been shown to increase the presence of the radioresistant CSC population.

The use of carbon ions could theoretically overcome the radioresistance of CSCs due to the higher RBE and increased LET, and cytotoxic effects of carbon that are independent of hypoxia. Bao *et al*. showed that the fraction of CSCs in glioma in fact increased after the administration of photon RT [73]. Additionally, this population showed a survival advantage compared to the non-CSCs. Subsequently, they found that the observed radioresistance was related to the DNA damage response, where CSCs were more readily able to repair DNA damage as compared to their non-CSC counterparts. Again, this represents a scenario where the use of carbon ions could potentially eliminate radioresistance as the formation of clustered damage is less amenable to repair.

Masunaga *et al*., showed that a pimonidazole-unlabelled subfraction of quiescent tumor cells, considered the closest representative subpopulation to CSCs, may be a critical target in tumor control [76]. Treatment with carbon ions were shown to decrease the difference in radiosensitivity between quiescent and non-quiescent cells, as well as, hypoxic and normoxic cells.

Tumor markers such as, CD133 and CD44, and other assays (e.g. side population assay) are being used to identify subpopulations of CSCs. However, these methods are laden with challenges, as there is no standard CSC marker [79-81]. Also, certain non-CSCs may contain some or all of the CSCs characteristics. If a consensual agreement of CSC identification can be achieved, it can be incorporated into the decision to use carbon ion therapy. A significant therapeutic benefit could be elicited when using carbon ions in patients shown to harbor a large subpopulation of CSCs.

### Tumor microenvironment and metabolism

The interplay between tumor metabolism and microenvironment play a critical part in establishing the radioresistant phenotype by working in conjunction with, or even affecting the classical and neo-radiobiologic factors. Understanding and characterizing the tumor microenvironment has recently become quite popular, as it is postulated to play a large role in tumor invasiveness, metastasis, maintenance, and recovery of the tumor bulk and blood supply [82,83].

The microvasculature response to irradiation varies over the range of doses given, and with current standard fractionation schedules (1.5 to 2 Gy per fraction) the effect on the microenvironment may be having the opposite effect than desired. With current fractionated radiotherapy, the microenvironment, especially the microvasculature is protected by the action of HIF-1. HIF-1 is also responsible for vascular protection, reestablishment of tumor blood and nutrient supply, and post-irradiation recurrence [40]. It also has been shown clinically, in various cancers that may benefit from carbon ion RT, to correlate with poor LC and increased mortality [84-86]. Upregulation of HIF-1 induces the tumor cell to produce VEGF, amongst other proangiogenic factors, inducing angiogenesis and vasculogenesis, along with other cellular mechanisms, that protect the microenvironment from radiation-induced endothelial apoptosis [87].

In addition to the secretion of VEGF, the secretion of stromal-derived factor 1 (SDF1) is upregulated. Combined VEGF and SDF1 result in the recruitment of bone marrow-derived cells that promote neovascularization and stimulate the regrowth and survival of tumor cells [88,89]. Further evidence suggests that carbon ion RT may suppress the production of x-ray induced angiogenesis mediators, and in doing so increases radiosensitivity [90].

With further exploration of this relationship, significant benefit in other cancers that have shown a similar relationship to angiogenesis and response to anti-VEGF therapy, such as gliomas, could prove worthwhile. It seems logical that tumors with high HIF-1 expression may see markedly increased radiosensitivity. Notably, however, not all tumor types express HIF-1α, therefore, inhibition of HIF-1 may be more effective if combined with carbon ion therapy imparting lasting deterioration of the microvasculature, affecting many critical pathways (e.g. angiogenesis, neovasculogenesis, and glucose metabolism) downstream, possibly resulting in enhanced patient outcomes.

### Glucose metabolism

A hallmark of cancer cells is the high rate of glucose consumption and lactate production regardless of oxygen tension, known as the Warburg effect [91,92]. Under aerobic conditions normal cells generate energy (ATP) by processing glucose both through glycolysis (inefficient) and mitochondrial oxidation (more efficient). However, hypoxia decreases the rate of mitochondrial oxidation causing the activation of a glycolytic switch causing tumor cells to produce energy using glycolysis, a process known as the Pasteur Effect or anaerobic glycolysis [93,94].

It is interesting that tumor cells use the less efficient glycolytic pathway for energy production regardless of oxygen tension; however, this pathway actually serves multiple purposes that enable tumor growth and proliferation (Figure 2). Various hypotheses exist as to why this phenomenon exists, with two revolving around the mitochondria. One hypothesis is that the cell actively tries to avoid the mitochondria for its survival, as it is the organelle responsible for initiating apoptosis through various cascades of caspases. The second: tumor cells have damaged and permeable mitochondrial membranes that reduce the efficiency of mitochondrial oxidative phosphorylation (reviewed in [95]).

Additionally, the glycolytic products lactate and pyruvate induce HIF-1α accumulation, which in turn initiate the transcription of transporters and enzymes that regulate glycolysis and the pentose phosphate pathway [40]. Furthermore, glucose-6-phosphate is incorporated in the pentose phosphate pathway. This pathway is responsible for synthesizing precursor macromolecules necessary for tumor growth and proliferation [40]. When cells do not need these macromolecules, the intermediates of the pentose phosphate pathway, fructose-6-phosphate and glyceraldehyde-3-phosphate, are recycled back into glycolysis to produce pyruvate and lactate, ergo continuing the cycle.

Tumor cells face direct and indirect mechanisms of damage from radiotherapy, notably the indirect action of radiation-induced radicals and oxidative stress. To counter these, cells upregulate their endogenous antioxidant capacity by accumulating the glycolysis metabolism products: pyruvate, lactate, and the redox couples glutathione (GSH)/glutathione disulfide and NAD(P)H/NAD(P)+, which work as a buffer network that scavenges free radicals and reactive oxygen species [96-99]. Moreover, tumor glucose metabolism is involved in the synthesis of these reducing species, which protects DNA from free radical-mediated damage [96]. As carbon ions induce direct action on DNA, and do not produce radicals and oxidative stress, the concentration of glycolytic products would decrease, theoretically reducing radioresistance.

The targeting of tumor glucose metabolism has been shown to be an effective means of overcoming radioresistance in many tumor histologies [100-102]. Disrupting lactate efflux via monocarboxylate transporter (MCT) inhibition has been shown to enhance radiosensitivity in human glioma cells. Gliomas are highly glycolytic producing large amounts of lactate; when lactate efflux was blocked by α-cyano-4-hydroxycinnamic acid (ACCA) the levels of intracellular lactate and GSH decreased, and enhanced radiosensitivity [100].

Intriguingly, targeting GSH itself has also been shown to enhance radiosensitivity, however, only when combined with carbon ion therapy. Depleting GSH via combined dimethylfumarate and L-buthionine sulfoximine and carbon ion, prevented the transmission of chromosomal aberrations (complex rearrangements, chromosome breaks and losses) in the head and neck SCC cell lines SQ20B and SCC61, which are radio-resistant and -sensitive respectively [103]. This phenomenon was not seen in cells irradiated with X-rays. GSH depletion with carbon ion therapy may give a considerable survival advantage to the patient as this therapy appears to minimize genomic instability and may enhance LC.

### Iron metabolism

Iron metabolism through the dysregulation of the Iron Regulatory Protein (IRP) 1-mediated pathway has recently been shown to induce radioresistance in HL60 human myeloid leukemia cells to low LET, specifically γ-radiation [104]. Iron is one of the most reactive metals in cells, and is incorporated by a plethora of enzymes as a co-factor. Due to the high reactivity, iron is able to undergo Fenton and Haber-Weiss reactions with hydrogen peroxide yielding ferric iron (Fe^3+^), hydroxide, and the highly damaging hydroxyl radical (·OH) [105,106].

To prevent these reactions from taking place, mammalian cells have evolved to develop a rapid response iron sequestration system mediated primarily through IRP1 and 2 [107]. An increasing body of evidence points towards the association between radioresistance and substantially reduced protein oxidation immediately following irradiation in lower organisms. Through the knockdown of IRP1 via short-hairpin RNA, Haro *et al*. found that human myeloid leukemia cells were more resistant to low LET radiation [104]. Furthermore, knockdown of IRP1 led to more rapid DNA DSB repair and reduced protein oxidation, thus the claim can be made that control of intracellular iron could be a novel radioresistance mechanism [104].

Interestingly however, when these same cells were subjected to high LET radiation (α-particles), their clonogenic survival and overall radiosensitivity remained unaffected. This may be partly explained by the complexity of DNA damage that high LET radiation directly imparts. Additionally, cells have been observed to have become increasingly less dependent on apoptosis in overall cell death [10].

Cumulatively, these data can be used as positive support for treating malignancies with carbon ions, and it is evident that tumor metabolism and the tumor microenvironment are critical players in conferring the resistant phenotype; yet more studies need to be done to fully understand the interplay between the aberrant tumor metabolism and microenvironment, and their impact on the mechanisms of radioresistance. Furthermore, it is still unclear whether radiosensitizers are necessary for enhanced patient benefit with carbon ion therapy. Some evidence appears to be indicating that targeting the tumor metabolism and/or microenvironment with inhibitors could augment cancer cell death, leading to the hypothesis that their effect may be augmented when combined with the high LET carbon ion beam.

### Anatomical factors

The presence of significant sensitive structures adjacent to the tumor mass can play a substantial role in selecting appropriate treatment modalities. Anatomy can be the limiting factor in both surgical resection and irradiation due to the potential for sequelae and morbidity. Typical anatomic constraints are: 1) the presence of nerves adjacent to the tumor whose integrity could be compromised as a result of surgery or radiation, 2) the inability to resect the tumor with negative margins while preserving important structures, and 3) the inability to radiate to a curative dose without overdosing the organs at risk in the field, undermining the dose that can be safely prescribed to the tumor.

**Figure 3:**
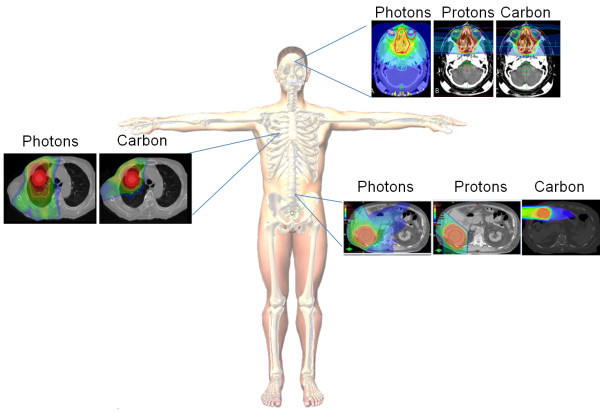
**Anatomical constraints can be overcome with carbon ions for various histologies.** Comparing the same histologies at different sites which have anatomical constraints such as glioblastoma multiforme (intracranial), lung (thoracic region), and rectal carcinoma (abdominal/pelvic) using treatment planning software for photons, protons and carbon it is evident that implementing carbon ions gives better biological dosage to the target area (tumor) while limiting treatment to surrounding healthy tissue. Adapted with permission from [169-172].

While intensity-modulated radiation therapy (IMRT) can often accomplish sparing of adjacent structures using low LET irradiation, it is associated with a much higher integral dose. As carbon ions exhibit a Bragg peak, they enable the delivery of radiation to the tumor while decreasing the dose delivered to adjacent organs at risk. Moreover, carbon ions have a decreased lateral penumbra, thus enabling better dose accuracy (Figure 3). Consequently, a number of clinical trials have been carried out with tumors in areas with anatomic constraints (Reviewed in [21,108]) (Table 1).

**Table 1:** **Effectiveness comparison for various histologies by anatomical location between Standard of Care** (**SOC**) **and Carbon Ions**

**Site**	**No. of carbon ion studies**	**5-year LC range**		**Toxicity range (late** ≥ **GIII injury)**		**References**
		**SOC**	**Carbon**	**SOC**	**Carbon**	
***Intracranial***						
**Glioma**	2	< 20%	-	Location dependent	-	Trials ongoing^§†^
**Meningioma**	2	80-90%	-	Location dependent	-	Trials ongoing^§‡^
***Head and Neck***						
**Adenoid cystic**	3	27-72%	26-96%	0-12.9%	0-17%	[141,142]
**Bone/soft tissue sarcoma**	2	43-70%	24-73%	0%	2-18.5%	[20,140,143-147]
**Skull base**	3	46-73%	82-88%	0-7%	0-5%	[117-121,148]
***Thorax***						
**NSCLC**	4	80-97%	90-95%	0-15%	3% (pneumonitis)	[21,149]
***Abdomen and Pelvis***						
**HCC**	4	75-96%	81-96%	7-22%	3-4%	[21,130-133,150]
**Pancreas**	2	10-20%	66-100%	1.8-20%	7.7%	[136,151-153]
**Prostate**	2	80-95%**	87-99%*	4-28%	0.1-25%	[21,24,154-159]
**Rectal cancer**	1	24-28%	95%	14-27%	-	[21,160-162]
**Cervix cancer**	1	20%	53%	0-10.6	9.6-18.2%	[163-165]
**Sacral chordoma**	1	55-72%	88%	17.6%	5.9%-17.9%	[166-168]
**Chondrosarcoma**	1	20-40%	60%	-	-	[167,168]

*Abbreviations:SOC* Standard of Care, *LC* Local Control, *HCC* Hepatocellular carcinoma, *GIII* Grade III toxicity, *OS (Overall survival); **bPFS (biochemical progression free survival); ^§^CLEOPATRA (NCT01165671); ^†^CINDERELLA (NCT01166308); ^‡^MARCIE (NCT01166321).

### Intracranial tumors

Two of the most common intracranial tumors that have received attention for carbon ion RT are gliomas and meningiomas. Gliomas, the most common form of primary brain cancers, account for nearly 51% of all central nervous system tumors [109]. GBM, a WHO Grade IV glioma, has a median OS of approximately nine months and has characteristically been described as radioresistant [109-112]. Typically, maximal safe resection is first-line therapy, yet often it can be difficult due to various factors, such as the location of the tumor relative to critical structures (i.e. optic chiasm, white matter tracts, ventricles, motor and lingual cortices etc.). Meningiomas are generally less aggressive, however, can have a very high recurrence rate, depending on the subtype, when treated with surgical resection alone; depending on their location resection may be impossible. Proximity of the tumor or tumor bed to the chiasm, optic nerves, or brainstem can make the administration of doses ≥ 54 Gy difficult as the dose tolerance to the chiasm and optic nerves is 56 Gy, and the dose tolerance to the brain stem is 54–60 Gy [113].

In both cases, sparing adjacent noninvolved brain is also a concern in terms of late toxicity and secondary malignancy, especially in younger patients with a more favorable prognosis. And despite the administration of curative intent doses to gliomas, these almost inevitably recur, usually on the order of up to two centimeters from the initial resection cavity [114,115]. For meningiomas especially, those tumors are adjacent to the skull base or are in close proximity to cranial nerves, thus rendering resection and RT challenging. The superior RBE of carbon ions and the ability to dose escalate while sparing organs at risk in the field may improve the prognosis of glioma patients while sparing toxicity for patients with a more favorable prognosis (i.e. meningioma).

Carbon ion treatment of these malignancies is the subject of multiple ongoing clinical trials (Table 1). For the treatment of glioma, when rationalizing the use of carbon ions, the emphasis is placed on improvement in LC, whereas, in the case of meningioma, LC can be obtained with conventional photon treatment. The emphasis here is placed on minimizing toxicity, an important consideration in discussing the design of future clinical trials (discussed below).

### Head/neck and thorax tumors

Tumors of the head and neck, orbit, skull base, or upper cervical spine present a therapeutic challenge to both surgeons and radiation oncologists due to their proximity to the oral cavity, pharynx, larynx, paranasal sinuses and nasal cavity, and salivary glands, as well as the cranial nerves and the brain stem. For a great proportion of these tumors, the extent of resection correlates with the likelihood of LC and outcome. The lowest dose limiting structure in this area is likely the eye lens, however; since cataract surgery has become increasingly common, it is likely that the salivary gland with a mean dose of 26 Gy for 20% risk of salivary dysfunction and xerostomia would be the lowest limiting dose that has the largest clinical impact [113].

While xerostomia is detrimental to quality of life and dentition, the brain stem and the spinal cord with the risk of developing long term sequelae can present significantly higher challenges to treating tumors with curative doses in this area. This is especially true for chordomas and chondrosarcomas that are in close proximity to the spinal cord. Chordomas are usually slow-growing, low-grade malignancies that can arise from the sacrum (50-60% of cases), skull base (25–35%), cervical vertebrae (~10%), and throacolumbar vertebrae (5% of cases) [116]. Regardless, surgery remains the primary modality for the treatment of chordomas, however due to the closeness of critical structures as mentioned above, it is often difficult to achieve. Radiation too is problematic, as a result of dose constraints.

A dose of 50 Gy to the spinal cord carries a 0.2% chance of myelopathy, whereas a dose of 60 Gy carries as 6% chance [113]. For the brain stem, a dose of less than 59 Gy to any 1 to 10 cc volume reduces the risk of neuropathy or necrosis to < 5% [113]. Generally, doses in the range of 60 to 70 Gy, are required to eradicate the gross disease in most malignant tumors with the exception of lymphoma. This dose can be difficult to administer safely to sites adjacent to the spinal cord or brain stem (e.g. chordoma, chondrosarcoma, bone and soft tissue sarcomas of the head and neck, and other locally advanced head and neck or spinal tumors not amenable to resection with negative margins). The sharp lateral fall off of carbon ions can help spare these structures, and may enable the administration of a higher dose to the tumor, thus improving LC. An improvement in LC has been observed in adenoid cystic carcinoma, bone and soft tissue sarcoma, and skull base and upper cervical tumors. A similar or better toxicity profile, as compared to, proton or photon treatment has also been shown in these sites (Table 1).

Achievement of wide negative margins appears to be correlated with the rate of local recurrence and survival, with recurrence rates near 70% when negative margins are not achieved [116]. For skull base and upper cervical spine tumors treated at NIRS, patients had a 5-year LC and survival rate above 80% with 5% of patients experiencing > Grade III toxicity in one study, and no patients experiencing Grade III toxicity in two other studies [117,118]. Not only did carbon ion treatment provide a similar or superior toxicity profile for chordomas, it also showed superior 5 and 10 year LC as compared to proton or proton/photon treatment [119-121] (Table 1).

Tumors originating from the thorax can be difficult to resect with negative margins. Furthermore, they can also be difficult to radiate due to lung and heart dose constraints. Meeting lung constraints can be achieved with using the field in field technique or IMRT; however, with both of these techniques, the mean lung dose often exceeds 14–15 Gy, increasing the likelihood of pneumonitis to > 15%. Additionally, it is often difficult to meet the traditional dose constraint of V20 < 30% (volume receiving 20 Gy to represent less than 30% of the total lung volume) [113]. The traditional dose constraint to the heart is V30 < 46% for < 15% risk of incurring pericarditis, however, the constraint for long term cardiac mortality, V25 < 10% for 1% risk of cardiac mortality is rarely ever achieved when attempting to treat to curative doses in the thorax [113].

In unresectable Stage I and II lung cancer, definitive radiation is an established treatment option, and while LC may be quite good (upwards of 85%), OS remains poor. SBRT is an increasingly common method to treat unresectable Stage I and II lung cancer, and has improved both the coverage of the tumor, as well as, decreased toxicity [122,123]. In larger tumors or those in close proximity to the mediastinum, SBRT is often not possible to perform; it is these patients that may benefit from carbon ion treatment [124-126]. Stage I patients with tumors > 3 cm (T2) have been treated with carbon ions and found that carbon provided superior dose distribution while treating less normal tissue, and the rate of radiation pneumonitis was 3% (Table 1). Higher toxicity was observed in patients treated prior to 2006 when only 1 to 2 portals were used in treatment.

### Abdominal and pelvic sites

Hepatocellular carcinoma (HCC) is the third leading cause of cancer mortality worldwide and accounts for nearly 90% of primary liver cancers in the United States [127]. The resection of HCC tumors is a major procedure, especially in high-risk patients, with post-operative death rates between 5 and 20% [128,129]. The prognosis and outcome of HCC is generally poor, with only 10 to 20% of HCC tumors able to be successfully resected with wide negative margins; the 5-year survival rate is close to only 15% [21].

Traditionally, the treatment of liver tumors with external beam RT has been limited by the dose constraint to the liver, with a mean dose of 30 to 32 Gy for a < 5% risk of development of radiation induced liver dysfunction (RILD). This is often problematic as the capacity of the liver to tolerate radiation in these patients may be undermined by significant liver impairment prior to the administration of RT. Currently, with the exception of radiofrequency ablation; the administration of RT remains largely a palliative modality as curative doses cannot be administered. Some evidence suggests that localized tumors > 5 cm may benefit from carbon ion RT [130]. Four studies have investigated the administration of carbon ion treatment for HCC (Table 1) with promising results [21,131-133].

Pancreatic cancer is the fourth leading cause of cancer mortality in the United States, with a 5-year OS rate of at most 5% [134]. Resection for ductal pancreatic adenocarcinoma, the most frequent pancreatic malignancy, offers the only curative hope for patients and gives a significantly improved prognosis of 14 to 20 months, and a 25% 5-year survival rate [135]. Yet, the prognoses for pancreatic cancer remain poor for unresectable tumors, with median survival around 4 to 8 months [134]. RT to this area with curative intent is not possible due to doses to organs at risk in the field which include liver (discussed above), small bowel (with general dose constraint: max point dose of 45 Gy or QUANTEC dose constraints of V15 < 120 cc and V45 < 195 cc for a 10% risk of ≥ Grade III toxicity), and stomach (D100% < 45 Gy for risk of ulceration of 7%). Typically, however, the field that would have to be treated postoperatively is exceedingly large that the administration of even microscopic disease doses is often difficult.

The superior depth dose distribution of carbon ions makes this modality attractive for pancreatic cancer from an anatomical perspective, while the superior RBE may make response even more likely in selected patients. Carbon ions have been employed both pre- and postoperatively with favorable toxicity profiles [136,137]. The combination of carbon ion treatment with Capacitabine chemotherapy is the subject of ongoing clinical trials PHOENIX (NCT01795274), as well as, treatment volumes and movement management (KFO 214).

If the standard treatment for rectal cancers, concurrent chemo-radiation followed by surgical resection, is done, postoperative pelvic recurrences are rare. Yet, if only surgical resection is done the incidence of recurrence is still around 5 to 20% [21]. The curative intent option for the management of a pelvic recurrence is often a total pelvic exenteration. This surgery is highly invasive and dramatically decreases the patient’s quality of life. The resection rate for locally recurrent colorectal cancers has been reported to be between 3% and 30%; with a majority of these patients ineligible for resection they are subsequently referred for RT [138]. The dose to the cord, small bowel (discussed above), and kidneys (mean < 15–18 Gy for < 5% clinical dysfunction,) in addition to, the need to achieve as much sparing as possible of at least one of the kidneys makes the administration of RT challenging. Here again the superior depth dose distribution of carbon ions may make curative intent treatment or even retreatment possible. The treatment of recurrent rectal cancer with carbon ion is the subject of one trial (Table 1), which reports very good LC (upwards of 95%); however, toxicity results are scant and long term results are not yet available. However, it is the subject of an ongoing clinical trial aimed at determining the optimal dose and PFS, as part of the phase II component of the trial [139].

When taking into account the anatomic barriers in administrating RT, some of the dose constraints have, at least partially, been overcome by IMRT, SBRT, or proton treatment. With the exception of proton treatment, the integral dose with these techniques is higher as compared to conventional plans. Both anatomic and integral dose constraints may have been overcome further by proton treatment. This however, does not have the benefit of a superior RBE. We feel that the majority of the sites discussed here with significant anatomical constraints, would fare well with continued exploration of carbon ion treatment due to: (1) superior depth dose distribution which it shares with protons, and (2) a superior RBE which may significantly improve tumor control. Long term clinical data will be necessary to make a complete assessment of optimal histologies; and short and long term toxicities, as well as, optimal dose/fractionation schemes will be necessary. Accrual of these data is the subject of ongoing trials (Table 1).

### Future clinical trial design

Currently, clinical trial design is based on the assumption that the same biologic effective dose is administered in the photon, proton and carbon ion arms. Additional knowledge of how to equate these doses is necessary, breaking away from the traditional referencing to photon doses. To help improve understanding and clinical trial design knowledge of the responses of various histologies, in addition to, early and late responding normal tissues to different radiation particles over a range of doses needs to be increased.

It is imperative to look at differences in RBE and tissue type in order to create the best therapeutic ratio. The response in cells and tissues are likely to be different, and certainly tissue dependent when administering large doses of carbon ion therapy, as compared to the traditional photon therapy fractionation 1.8 to 2.0 Gy/fraction. These differences may translate into different degrees of damage in the vascular structures of late responding normal tissue or, ideally, in the tumor stroma and tissue. Future clinical trial design should be aimed at exploiting the differences in radiosensitivity between cells radioresistant to low LET RT and sensitive to carbon ion RT to enable (1) adequate selection of histologies, and (2) adequate selection of patients most likely to benefit from this modality based on biomarkers and imaging.

**Figure 4:**
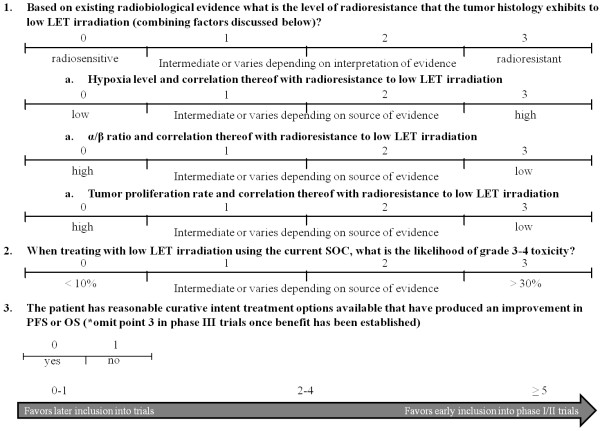
**Grading scale of histologies to warrant carbon ion exploration in future clinical trials.** Grading scale that should be used to select patients and tumor histologies to determine inclusion earlier or later in clinical trials.

Clinical trial design involving carbon ion therapy should proceed as one would if involved in any other therapeutic intervention along phase I trials, proceeding into phase II, and subsequently phase III, understanding that this logical progression may at times require the combination of phases to advance the field. Phase I trials have occurred in a significant proportion of the tumors discussed in this paper [21,140]. The selection of patients and tumor histologies should ideally occur along the lines describes in Figure 4, recognizing that none of the measures, whether hypoxia, α/β ratio, or tumor proliferation, may be fully reflective of the tumor microenvironment and true radiosensitivity of the tumor.

The addition of tumor biomarkers, that are as yet unidentified, that may predict response to carbon ion therapy should be incorporated into the decision algorithm once available. A cumulative score (Figure 4) of ≥ 5 would essentially describe a tumor radioresistant to low LET irradiation, with the standard of care (SOC) treatment causing significant toxicity. It would also include patients who have no curative options available. Tumor histologies and patients with a score of 0–1 derive significant benefit from current SOC, and thus would only become candidates for trials with carbon ion technology once significant OS or PFS benefit has been obtained in randomized trials, when compared to current SOC (i.e. once benefit is seen over and above that with low LET radiation in randomized trials, the technology may then be extrapolated to additional sites that already do well with current SOC in the hope of deriving additional benefit or decreasing toxicity).

To a great extent, the currently available phase I/II trials follow these guidelines. Once promising results have been obtained, as is currently the case for a number of sites, phase II trials can be advanced to randomized phase III trials where carbon ion treatment should be compared with current SOC for that histology or site. An additional arm could explore the addition of a systemic, concurrently administered, agent with carbon, when the same agent is part of SOC when using low LET irradiation.

A significant concern in proceeding with the comparison to current SOC is the problem of the carbon ion RBE, and its comparison to the RBE of low LET radiation. It is likely that further preclinical and clinical data are required before a sound comparison can be made. Although this describes the ideal way to introduce carbon ion technology into SOC, it is unlikely that the natural progression of phase III trials will occur this way, as equipoise may have been disturbed sufficiently prior to their introduction, thus making the acquisition of patients in such protocols unrealistic. However, since carbon ion technology is expensive, and as a result difficult to acquire, stringent control can be exercised thus ensuring that patients will not be treated outside of established protocols in order to advance the field and improve patient outcomes.

## Conclusions

**Figure 5:**
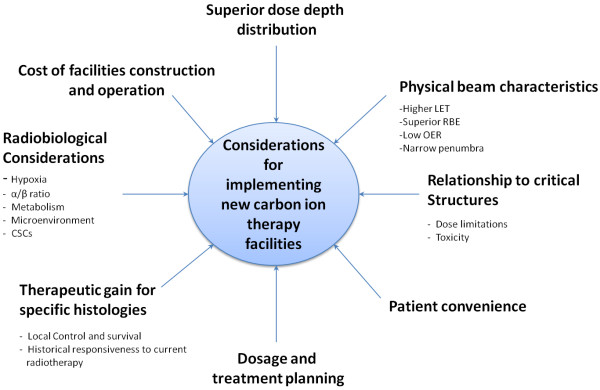
**Considerations for the implementation of new carbon ion facilities.** When beginning the process for proposing the construction of new carbon ion facilities, the decision is multi-factorial and a range of considerations must be considered, from patients, to treatments, to cost. Carbon ion RT is an exciting new field, however, in its infancy, and needs to be implemented with caution only when there is a sound knowledge base of its understanding.

In summary, carbon ion therapy is recommended for tumors, some of which are described here, that are radioresistant and/or located close to critical structures. The use of carbon ion therapy is sensible, when the advantages of using carbon ions outweigh the therapeutic advantages that can already be obtained with fractionated photon RT. Future clinical trials should be aimed at the comparison of photon, proton and carbon ion treatment in conjunction with the identification of molecular biomarkers of hypoxia, and metabolism, in an effort to achieve optimal patient selection (Figure 5). With the dawn of personalized medicine, those tumors that have traditionally responded well to other radiation species should continue to be treated with those species, while the rare or non-responsive malignancies should be treated with carbon ions in a patient specific manner. Furthermore, the expansion of carbon ion treatment facilities should be undertaken in the United States.

### Disclaimer

The views expressed in this article are those of the author and do not necessarily reflect the official policy or position of the Department of the Navy, Army or Air Force, the Department of Defense, nor the U.S. Government.

## Abbreviations

LET: Linear energy transfer; LC: Local control; RT: Radiation therapy; HILAC: Heavy Ion Linear Accelerator; HIMAC: Heavy Ion Medical Accelerator; WRNMMC: Walter Reed National Military Medical Center; HIT: Heidelberg Ion-Beam Therapy Center; RBE: relative biological effectiveness; SLDR: sub-lethal damage repair; LQ: Linear quadratic; SF2: Survival fraction at 2 Gy; OS: Overall survival; OER: Oxygen enhancement ratio; ODD: Oxygen dependent degradation domain; HIF: Hypoxia inducible factor; HRE: Hypoxia response element; VEGF: Vascular endothelial growth factor; IHC: Immunohistochemistry; MRI: Magnetic resonance imaging; PET: Positron emission tomography; GBM: Glioblastoma multiforme; DNA: Deoxyribonucleic acid; Tpot: potential doubling time; SCC: Squamous cell carcinoma; CSC: Cancer stem cell; SDF1: Stromal derived factor-1; MCT: Monocarboyxlate transporter; ACCA: α-cyano-4-hydroxycinnamic acid; GSH: Glutathione; IRP: Iron regulatory protein; IMRT: Intensity-modulated radiation therapy; NIRS: National Institute of Radiological Sciences; SBRT: Stereotactic body radiation therapy; RILD: Radiation induced liver dysfunction; HCC: Hepatocellular carcinoma; PFS: Progression free survival; SOC: Standard of care.

## Competing interests

All authors have no competing interests to declare.

## Authors’ contributions

CDS analyzed and interpreted the data; carried out the writing of the manuscript and figure preparation. AK made substantial contribution in the conception, analysis and interpretation of the data, as well as writing of the manuscript and figure preparation. AB assisted in the drafting of the manuscript. JJO’C assisted in the drafting of the manuscript. KAC provided general supervision and assisted in the design, analysis and writing of the manuscript and figure preparation. All authors read and approved the final manuscript.
